# The protective role of *Artemisia annua* against chlorpyrifos toxicity in Nile tilapia

**DOI:** 10.1038/s41598-026-47713-1

**Published:** 2026-04-17

**Authors:** Aya Ashry, Ehab Yahya Abdelhiee, Adel Hassan Saad, Mohammed Morsi Elkamshishi, Safaa E. Abdo, Sabreen E. Fadl

**Affiliations:** 1 Biochemistry Dept, Faculty of Veterinary Medicine, Matrouh University, Matrouh, Egypt; 2 Forensic Medicine and Toxicology Department, Faculty of Veterinary Medicine, Matrouh University, Matrouh, 51744 Egypt; 3 Nutrition and Clinical Nutrition Department, Faculty of Veterinary Medicine, Matrouh University, Matrouh, 51744 Egypt; 4 Department of Animal, Poultry Hygiene and Zoonoses, Faculty of Veterinary Medicine, Matrouh University, Matrouh, Egypt; 5 Genetics and Genetic Engineering, Department of Animal Wealth Development, Faculty of Veterinary Medicine, Kafrelsheikh University, Kafrelsheikh, Egypt

**Keywords:** Artemisia annua, Chlorpyrifos, Gene expression, Pathology, Biochemistry, Diseases, Environmental sciences, Zoology

## Abstract

The adverse effect of chlorpyrifos (CPF) in fish farms is well known. However, dietary supplementation of *Artemisia annua (A. annua)* may decrease the negative effect of chlorpyrifos because of the ameliorating effect of *A. annua*. The aim of this study was to see how *A. annua* ameliorated the toxic effects of CPF on fish growth, health, growth-related gene expression, and gills, liver, and intestinal histology for 30 days. 120 Nile tilapia fish were divided into four groups according to *A. annua* with/without CPF. The Cont group was fed a formulated basal diet without CPF water contamination. The Art group was fed on a diet supplemented with *A. annua* and without CPF-contaminated water. The CPF group was fed a formulated basal diet with CPF water contamination. The CPF + Art group was fed on a diet supplemented with *A. annua* and with CPF-contaminated water. The results of the study indicated that the mortality rate increased in the CPF group with obvious clinical signs and postmortem lesions. Growth parameters of Nile tilapia of CPF group (final body weight = 26.43 ± 0.98, weight gain = 5.07 ± 0.52, and feed intake = 11 ± 0.5) significantly (*P* ≤ 0.05) decreased when compared with the Cont group (final body weight = 33 ± 0.58, weight gain = 11.27 ± 0.37, and feed intake = 18.8 ± 0.85). Moreover, CPF-contaminated water led to a liver injury that was indicated by serum activities of ALT and AST (29.67 ± 0.67 and 86.67 ± 0.88, respectively) and decreased serum proteins (total protein = 3.04 ± 0.01, albumin = 1.02 ± 0.01, and globulin = 2.02 ± 0.01) when compared with the control group. The pathological lesions were observed in the gills, liver, and intestinal tissue, elucidate the ameliorative effect of Art. On the other side, the growth hormone receptor (*GSH*) expression wasn’t affected by the chlorpyrifos toxicity. While the Insulin-like growth factor-1 (*IGF1)* expression was downregulated by the chlorpyrifos toxicity. However, this negative effect is ameliorated by *A. annua* supplementation. The ameliorated effect of *A. annua* is demonstrated by the improvement of growth performance parameters, growth-related gene expression, biochemical markers, and histopathology of the different organs of Nile tilapia.

## Introduction

Over the past decade, Nile tilapia (*Oreochromis niloticus*) farming has experienced rapid expansion, with an annual growth rate of approximately 12.2%^1^. In Egypt, Nile tilapia is a crucial and economically significant species for aquaculture. The sector largely depends on agricultural drainage water, which increases the risk of water contamination from pesticides like chlorpyrifos^2^.

Chlorpyrifos (O, O-diethyl-O-3,5,6-trichlor-2-pyridyl phosphorothioate; CPF) is a versatile organophosphate insecticide widely used in agriculture to manage insects that damage crops. CPF can enter nearby water bodies through air drift or surface runoff^3^. It has been shown to impact various biological aspects in fish, including alterations in hematological profiles, histopathological changes, hepatic dysfunction, growth impairments, neurobehavioral disruptions, reproductive physiology, and endocrine abnormalities. Additionally, it can induce oxidative stress, genotoxic effects, and cytotoxic damage in aquatic organisms^4^. However, there was a gradual decrease in weight gain percentage (WG %), specific growth rate (SGR), and survival rate of Nile tilapia with increasing chlorpyrifos toxicity^5^.

Scientists have examined how medicinal plants with antioxidant effects might help shield against the toxicity of insecticides^6^. *Artemisia annua* is belongs to genus *Artemisia L.* of family *Asteraceae* and tribe *Anthemideae*. It is a medicinal plant distinguished by its high biological efficacy and low toxicity (Soares et al.,, 2020). It is also a notable source of monoterpenes, sesquiterpenes, flavonoids, aliphatic compounds, coumarins, and essential oils, which have been documented to possess a spectrum of therapeutic activities, including anti-tumor, antioxidant, anti-fungal, anti-inflammatory, anti-protozoal, and cytotoxic properties^7^. This extensive range of bioactive compounds, combined with its rich nutrient and antioxidant profile, makes *A. annua* an excellent candidate for potential use as an herbal tonic for humans and a significant supplementary feed additive in livestock production systems (Brisibe et al.,, 2009). The effects of dietary enzymatically-treated *A. annua* L. in a low fish meal diet on growth, antioxidation, metabolism, and intestinal health of *Micropterus salmoides* were investigated (Dai et al.,, 2023). The study provides evidence that *A. annua* improves growth performance, antioxidant status, and intestinal health. These findings suggest that similar benefits could be expected for Nile tilapia when *A. annua* is incorporated into their diet.

This study aimed to examine the impact of dietary supplement containing *A. annua* on the growth and growth-related gene expression, liver tests, and histopathological conditions of certain organs in Nile tilapia (*Oreochromis niloticus*) exposed to chlorpyrifos toxicity^8^.

## Materials and methods

### Experimental design and diet

To fulfill the aims of this study, 120 monosex male Nile tilapia (*Oreochromis niloticus*), each weighing 22.5 ± 1.44 g, were utilized. The experimental fish were obtained from a local farm. These fish underwent two weeks of acclimatization prior to the trial. The fish were allocated into four distinct experimental groups, with each group further divided into three replicates, each containing ten fish housed in separate glass aquariums of dimensions 40 cm x 40 cm x 80 cm. The levels of ammonia and pH were measured twice weekly. Additionally, water parameters were maintained within the normal range for growing Nile tilapia fish. The results of the water were expressed as mean ± SD: The water pH was 7.2 ± 0.1, the temperature was 26 ± 2.5 °C, the dissolved oxygen concentration was 6.7 ± 0.3 mg L⁻, and the total ammonia content was 0.22 ± 0.1 mg L⁻. The experimental groups were organized as follows: the Cont (control) group was fed a formulated diet and maintained in chlorpyrifos-free water. The CPF group received a formulated diet while being exposed to water with chlorpyrifos (The CPF used was a commercial formulation of Pyrifos El Nasr (El Nasr Chemical Company, Abu-Rawash, Egypt, 480 g/L chlorpyrifos, O, O-diethyl-O-(3,5,6-trichlor-2-pyridyl) phosphorothioate, 48% active ingredient) at concentration of 15 µg/L as indicated by Dawood et al. (2020b)^9^.

The Art group was fed a formulated diet supplemented with *A. annua* (5 g/kg diet) and kept in chlorpyrifos-free water^10^. The fresh leaves of *A. annua* were collected from a private agricultural field (Sharaky Agricultural Field) in Sidi Barrani (at 31°36’12.2"N; 25°55’31.1"E), Matrouh Governorate, Egypt. Then, *A. annua* leaves were purified with distilled water. They were washed several times with distilled water to remove extraneous materials such as dirt, sand, and other impurities; dried at 70 °C until a determined weight; finely powdered; and kept at 4 °C until used to prepare a formulated diet, and the CPF + Art group was given a formulated diet with *A. annua* (5 g/kg diet) and cultured in water containing 15 µg/L chlorpyrifos^11^.

The formulated diet Table 1 was prepared to meet the nutritional requirements of *O. niloticus* as per NRC^12^ and was prepared biweekly according to the methodology of Shimeino et al.^9^,. Experimental procedures followed the guidelines established by Abdelhamid et al.^13^,. During the initial two-week acclimation period, the fish were fed a formulated diet at a rate of 3% of their body weight. Over the subsequent 30-day experimental phase, feeding occurred twice daily at 9:00 AM and 13:00 PM, six days a week. The aquariums were maintained with fresh, dechlorinated, and aerated water, which was partially replaced daily, and chlorpyrifos was calculated and added daily in accordance with the volume of water added. The experimental fish were monitored for postmortem, mortality, and clinical symptoms during a 30-day period^14^.

**Table 1:** Physical and chemical composition of the basal diet.

Formulation	Percentage
	%
Fish meal (60% cp.)	12
Soybean meal (44% cp.)	30
Gluten	8
Wheat bran	12.7
Rice bran	12.5
Yellow corn	12
Wheat flour	10
Fish oil	1
Soybean oil	1
Vitamin C	0.2
Vitamin premix (1)	0.15
Mineral premix (2)	0.15
Choline chloride 50%	0.3
Total	100
Crude protein	31.1
Lipid	8.58
Fibers	6.22
Ash	7.11
Gross energy (KJ/g)	19.23

1 Vitamin premix (1.5 kg of diet): vitamin A, 300 000 IU; riboflavin, 500 mg; pyridoxine HCL, 400 mg; cyanocobalamin, 1.2 mg; thiamin, 20 mg; menadione, 40 mg; folic acid, 130 mg; biotin,.

10 mg; a-tocopherol, 3000 IU; myo-inositol, 8000 mg; calcium pantothenate, 760 mg; nicotinic.

acid 200 mg; choline chloride 8000 mg; vitamin D, 40 000 IU. (Modified from Wang et al^15^.

2006).

2 Mineral premix (1.5 kg): ZnSO4 7H2O, 4 g; CaCO3, 215 g; KCL, 90 g; KI, 0.04 g; NaCl 40.

g; CuSO4 5H2O 3 g; CoSO4 7H2O, 0.02 g; FeSO4-7H2O, 20 g; MnSO4 H2O, 3 g; MgSO4.

7H2O, 124 g; Ca (HPO4)2 2H2O, 500 g; (Modified from David & Gatlin 1996)^16^.

### Growth measurements

Body weight (BW): The fish were weighed at the beginning of the experiment (W0) and then every two weeks after that. The change in live body weight was used as a growth indicator. Weight gain (G)= (Final body weight−Initial body weight)^17^.

Feed intake (FI): Diets were given daily at 9:00 a.m. and 13:00 p.m. The difference between the weight of the presented feed and the remaining portion was used to determine the feed intake for the two weeks. This amount was then divided by the number of fish in each aquarium and totaled for each two weeks^18^.

The total feed intake per aquarium was divided by the total body weight growth per aquarium to determine the feed conversion ratio (FCR)^19^.

### Blood biochemical analysis

Tricaine methane sulphonate (100 mg/L) was used according to Dawood et al.^20^ to anesthetize all fish before blood collection. A sterile syringe was used to draw blood at the end of the trial from the caudal vein of nine fish per group, three fish per replication. Following blood coagulation, the serum was separated by centrifuging for 15 min at 3000 rpm. After blood collection, the fish were euthanized in a clove oil bath (50 mL/L) (Barijesans Co., Kashan, Iran) and gently dissected to collect tissue samples.

The techniques outlined by Doumas et al.^21^, and Doumas et al.^22^, were used to measure the amounts of albumin and serum total protein, respectively. A mathematical calculation was made to determine the globulin content. A colorimetric method based on Reitman and Frankel’s (1957) technique was used to measure the activity of AST and ALT at a wavelength of 540 nm.

### Gene expression

Total RNA was extracted from liver tissue samples (3 samples/replicate) using Trizol (iNtRON Biotechnology, Inc., Korea). RNA integrity and quantity were inspected using agarose gel electrophoresis and nanodrop. The cDNA was synthesized using reverse transcriptase according to the kit procedures (2X RT Mix, Applied Biotechnology, Egypt).

Genes specific primers pairs (Table 2) were used for the assessment of some growth-related genes in Nile tilapia, including growth-related gene (*GHR*) and Insulin-like growth factor-1 (*IGF1*). The gene amplification was done using the qPCR (PikoReal, Thermoscientific, TCR0024), and 2x -Lo-Rox- SYBR green kits (Applied Biotechnology, Egypt). The amplification conditions and the reaction mixture preparation were according to Hamed et al.^23^,. The annealing temperature was at 60 °C for 30 s. The relative mRNA expression was calculated as a fold change according to Livak and Schmittgen^24^. The elongation factor-1α (ef-1α) was used as an internal control gene. The fish group fed on the basal diet (without toxin or dietary supplementation) was the control group.

**Table 2:** The list of used primers.

Gene	Primer	Bp	Accession	Amplification efficiency (%)	Reference
*GHR*	F: CAGACTTCTACGCTCAGGTC R: CTGGATTCTGAGTTGCTGTC	80	AY973232.1	93.90	^25^
*IGF1*	F: GTTTGTCTGTGGAGAGCGAGG R: GAAGCAGCACTCGTCCACG	97	Y10830.1	94.45	
*Ef-1α*	F: TCAACGCTCAGGTCATCATC R: ACGGTCGATCTTCTCAACCA	125	XM_003458541	95.25	^26^

*GHR1*: Growth hormone receptor 1, *IGF1*: Insulin-like growth factor-1, *ef-1α*: Elongation factor 1 alpha.

### Histopathological examination

At the end of the 30-day experimental period, three euthanized fish were taken from each replicate (9 fish per group) to collect gills, liver, and intestinal samples from the freshly dead fish. These samples were fixed in 10% neutral buffered formalin for at least 24 h. The traditional paraffin embedding technique was then used on the fixed tissues. Afterward, 5-µm-thick sections were prepared from the paraffin blocks and stained with hematoxylin and eosin (H&E) for histological examination^27^. Intestinal morphometry was also analyzed, including villus length (from the tip of the villus to the crypt junction), villus width (measured at the median region, encompassing the two epithelial layers and lamina propria), and the number of goblet cells. Goblet cells were counted on similarly sized villi after being stained with PAS.

### Statistical analysis

The minimum sample size required for a research study was ascertained by power analysis prior to the experiment’s commencement. Additionally, in order to demonstrate homoscedasticity and normality, the data were subjected to the Shapiro-Wilk and Levene tests for normal distribution. The obtained data were statistically analyzed by one-way analysis of variance using SPSS to assess significant differences. A value of *P* < 0. 05 was considered to be significant.

## Results

### Clinical signs and postmortem lesions of the CPF toxicity

According to the clinical signs observed in the groups exposed to CPF toxicity, the affected fish exhibited a thick film of mucus covering their bodies, accompanied by abnormal movement and symptoms related to the neurological system. The treated groups with *A. annua* experienced a reduction in these symptoms. The dead fish from the CPF-contaminated groups that were not treated at the end of the experiment had pale gills with increased mucus secretion and internal organ congestion. The mortality rate was higher in the CPF group and decreased with treatment (Table 3). The mortality rates were 0 ± 0.0 ^c^, 16.66 ± 0.18^a^, 0 ± 0.0 ^c^, and 3.33 ± 1.22 ^b^ for Cont, CPF, Art, and CPF + Art groups, respectively.

### Growth performance

As mentioned in Table 3, The impact of *A. annua* on the growth performance of Nile tilapia under chlorpyrifos toxicity revealed significant (*P* ≤ 0.05) differences among the groups. The Cont group showed a higher final weight, weight gain, and feed intake compared to the chlorpyrifos-exposed group (CPF group). Additionally, FCR was higher in the CPF group in contrast to the groups of Cont and Art. However, the Art group exhibited a significant (*P* ≤ 0.05) increase in final weight, weight gain, and feed intake, outperforming all other groups with decreased FCR. Moreover, in terms of final weight, weight gain, and feed intake, the CPF + Art group demonstrated a significant (*P* ≤ 0.05) improvement compared to the Chlo group.

**Table 3:** Effect of dietary *A. annua* on growth performance parameters of Nile Tilapia under Chlorpyrifos Toxicity.

Groups Items	Cont	CPF	Art	CPF + Art
Initial weight (g)	21.73 ± 0.37	21.37 ± 0.47	21.93 ± 0.52	21.60 ± 0.83
Final weight (g)	33 ± 0.58 ^b^	26.43 ± 0.98^d^	38 ± 1.15 ^a^	29.73 ± 0.18 ^c^
WG (g)	11.27 ± 0.37 ^b^	5.07 ± 0.52 ^d^	16.07 ± 1.67 ^a^	8.13 ± 1.00 ^c^
Feed intake (g)	18.8 ± 0.85 ^b^	11 ± 0.5 ^d^	24.33 ± 2.60 ^a^	15.67 ± 2.33 ^c^
FCR	1.67 ± 0.02 ^c^	2.2 ± 0.15 ^a^	1.51 ± 0.01 ^d^	1.91 ± 0.05 ^b^

Values are means ± standard error. Mean values with different letters at the same row significantly differ at (*P* ≤ 0.05).

### Liver function tests

The liver test parameters revealed significant differences among the groups (Table 4). The Cont group displayed moderate protein levels and liver enzyme activity, while the chlorpyrifos-exposed group experienced a notable reduction in total protein, albumin, and globulin levels, coupled with an increase in the activities of the liver enzymes (AST and ALT), indicating liver damage.

However, the group supplemented with *A. annua* (Art group) showed a significant improvement in the albumin in contrast with the Cont group. However, the activity of ALT was higher in the Art group in contrast with the Cont group. The *A. annua* in chlorpyrifos-exposed fish led to an intermediate improvement, reducing the negative effects of chlorpyrifos toxicity.

**Table 4:** Effect of dietary *A. annua* on liver function tests of Nile Tilapia under Chlorpyrifos Toxicity.

Groups Items	Cont	CPF	Art	CPF + Art
T. Protein (g/dl)	4.39 ± 0.00 ^a^	3.04 ± 0.01 ^c^	4.31 ± 0.03^a^	4.10 ± 0.07 ^b^
Albumin (g/dl)	1.60 ± 0.05 ^b^	1.02 ± 0.01 ^c^	1.72 ± 0.02 ^a^	1.57 ± 0.01 ^b^
Globulin (g/dl)	2.80 ± 0.05 ^a^	2.02 ± 0.01 ^c^	2.59 ± 0.05 ^ab^	2.53 ± 0.08 ^b^
AST (U/L)	39.67 ± 0.33 ^c^	86.67 ± 0.88^a^	40.50 ± 0.29 ^c^	51.00 ± 0.58 ^b^
ALT (U/L)	12.00 ± 0.58 ^d^	29.67 ± 0.67 ^a^	15.50 ± 1.44 ^c^	18.00 ± 1.73 ^b^

Values are means ± standard error. Mean values with different letters at the same row significantly differ at (*P* ≤ 0.05).

### Gene expression

**Fig. 1:**
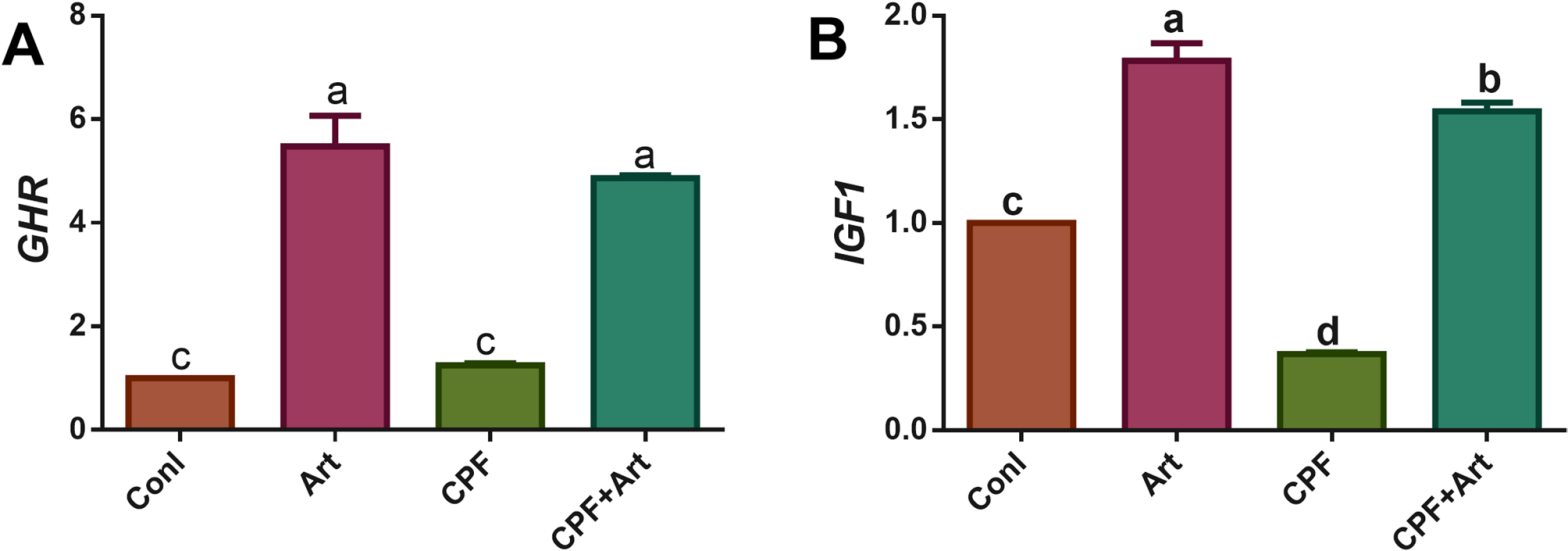
Expression of the growth-related genes in the liver tissue.

Figure 1 showed the expression of the growth-related gene (*GHR and IGF1*) of the various treated groups that were fed the control diet and the *A. annua* diet with/without chlorpyrifos. The expression of the *GHR* gene was upregulated in the Art groups (Art and CPF + Art) when compared with the other groups. However, the *GHR* expression wasn’t affected by the chlorpyrifos toxicity. While the *IGF1* expression was downregulated by the chlorpyrifos toxicity. However, the treatments upregulated the expression of the *IGF1*.

### Histopathology of the gills, liver and intestine

**Fig. 2:**
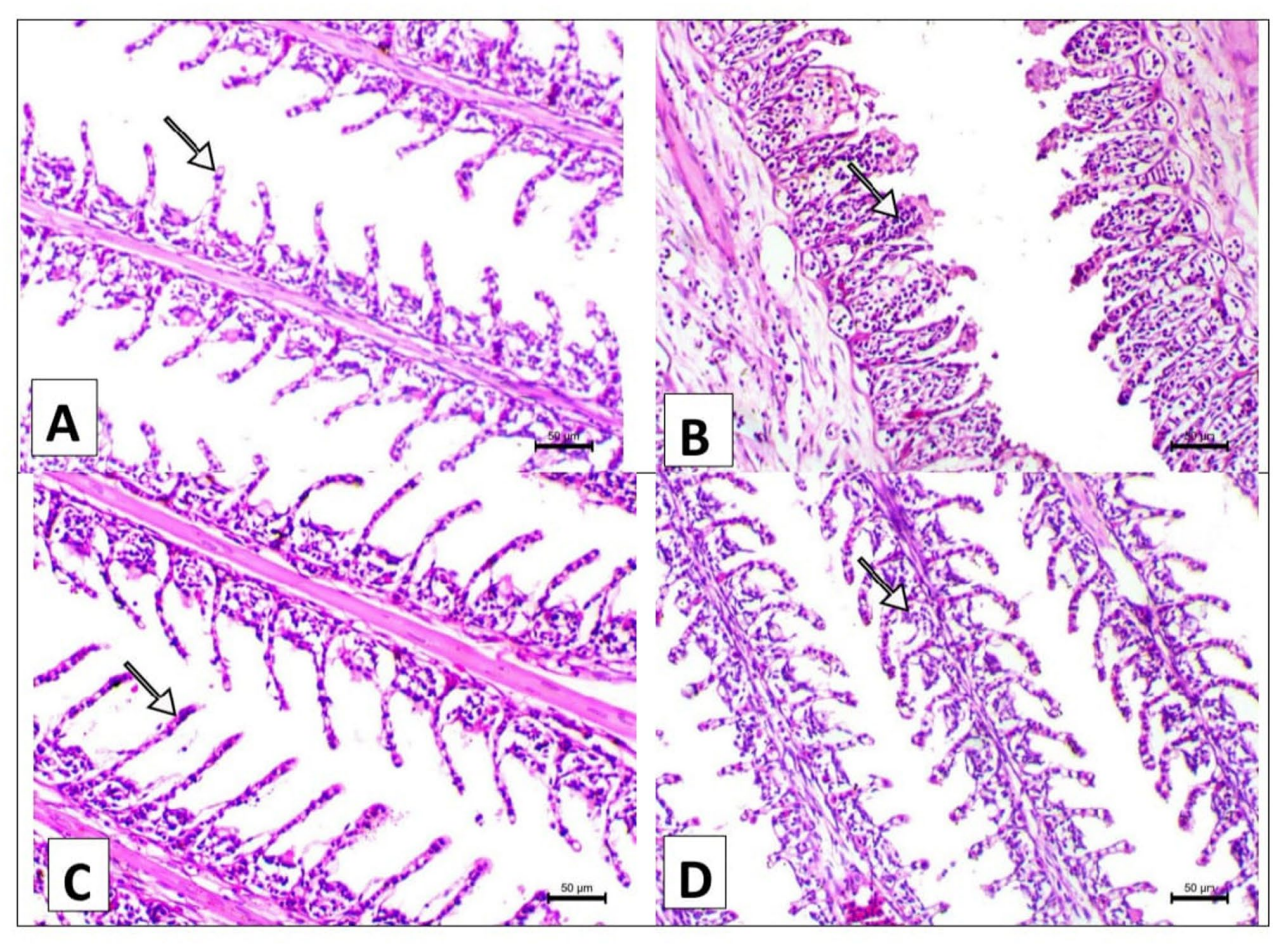
A. Gills section of fish supplemented with basal diet showing normal separated secondary lamellae (arrow). **B**. Gills section of fish intoxicated with chlorpyrifos in their water showing marked necrosis and blunting of the secondary lamellae associated with marked inflammatory cells infiltration (arrow), **C**. Gills section of fish supplemented with *A. Annua* showing normal long secondary lamellae (arrow), and **D**. Gills section of fish intoxicated with chlorpyrifos and supplemented with *A. Annua* showing remarkable decrease of lamellar thickening and adhesion of the secondary lamellae (arrow). H&E, X200 bar = 50 μm.

Figure 2 showed the histopathological changes of the gill tissues of the various treated groups that were fed the control diet and the *A. annua* diet with/without chlorpyrifos. The gills section of fish supplemented with a formulated diet or a formulated diet supplemented with *A. annua* showed normal separated secondary lamellae and normal long secondary lamellae, respectively (A and C). While the gills section of fish intoxicated with chlorpyrifos (CPF) in their water showed marked necrosis and blunting of the secondary lamellae associated with marked inflammatory cell infiltration (B). The gills section of fish intoxicated with chlorpyrifos and supplemented with *A. annua* (CPF + Art) showed a remarkable decrease of lamellar thickening and adhesion of the secondary lamellae (D).

**Fig. 3:**
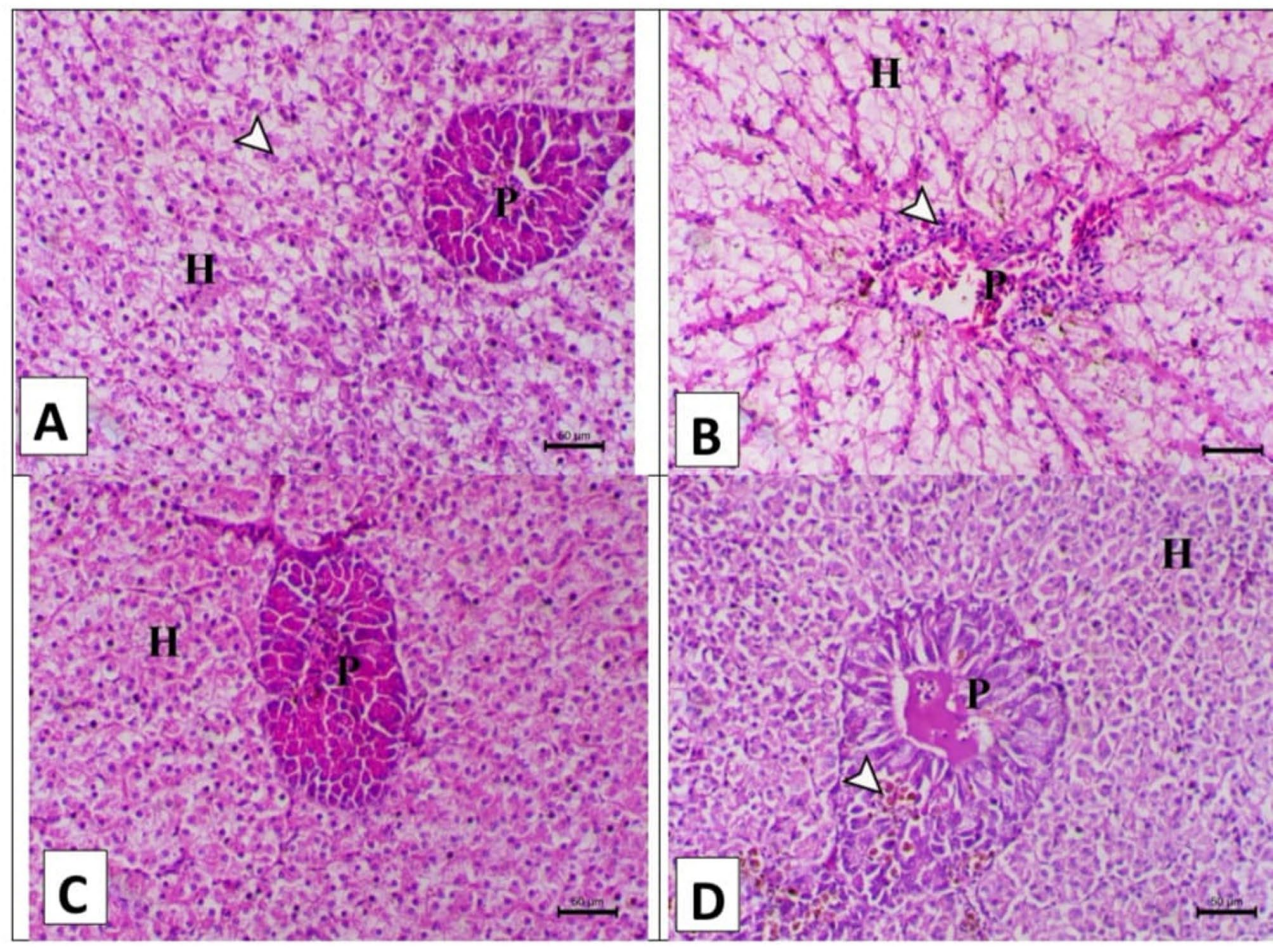
A. Hepatopancreas of fish supplemented with basal diet showing normal hepatic tissues (H) with mild vaculolation of the hepatocytes (arrowhead) and normal pancreatic acini (P) surrounding the central vien (arrow)., **B**. Hepatopancreas of fish intoxicated with chlorpyrifos in their water showing marked necrotic changes within the pancreatic tissue (P) associated with leukocytic cells infiltration (arrowhead) and marked vacuolation of the hepatocytes (H)., **C**. Hepatopancreas of fish supplemented with *Artemisia Annua* showing normal hepatic tissues (H) with esinophilic cytoplasm (arrowhead) and normal pancreatic acini (P) surrounding the central vien (arrow), and **D**. Hepatopancreas of fish intoxicated with chlorpyrifos and supplemented with *Artemisia Annua* showing normal hepatocytes (H) and with mild degenerative changes within the pancreatic acini associated with focal infiltration of haemosidrin pigment (arrowhead). H&E, X200 bar = 50 μm.

Figure 3 showed the histopathological changes of the hepatopancreas of the various treated groups that were fed the control diet and the *A. annua* diet with/without chlorpyrifos. The hepatopancreas of fish supplemented with a formulated diet showed normal hepatic tissues with mild vaculolation of the hepatocytes and normal pancreatic acini surrounding the central vein (A). Hepatopancreas of fish intoxicated with chlorpyrifos in their water showed marked necrotic changes within the pancreatic tissue associated with leukocytic cell infiltration and marked vacuolation of the hepatocytes (B). The hepatopancreas of fish supplemented with *A. annua* showing normal hepatic tissues with esinophilic cytoplasm and normal pancreatic acini surrounding the central vein (C). Hepatopancreas of fish intoxicated with chlorpyrifos and supplemented with *Artemisia annua* showed normal hepatocytes and mild degenerative changes within the pancreatic acini associated with focal infiltration of hemosiderin pigment (D).

**Fig. 4:**
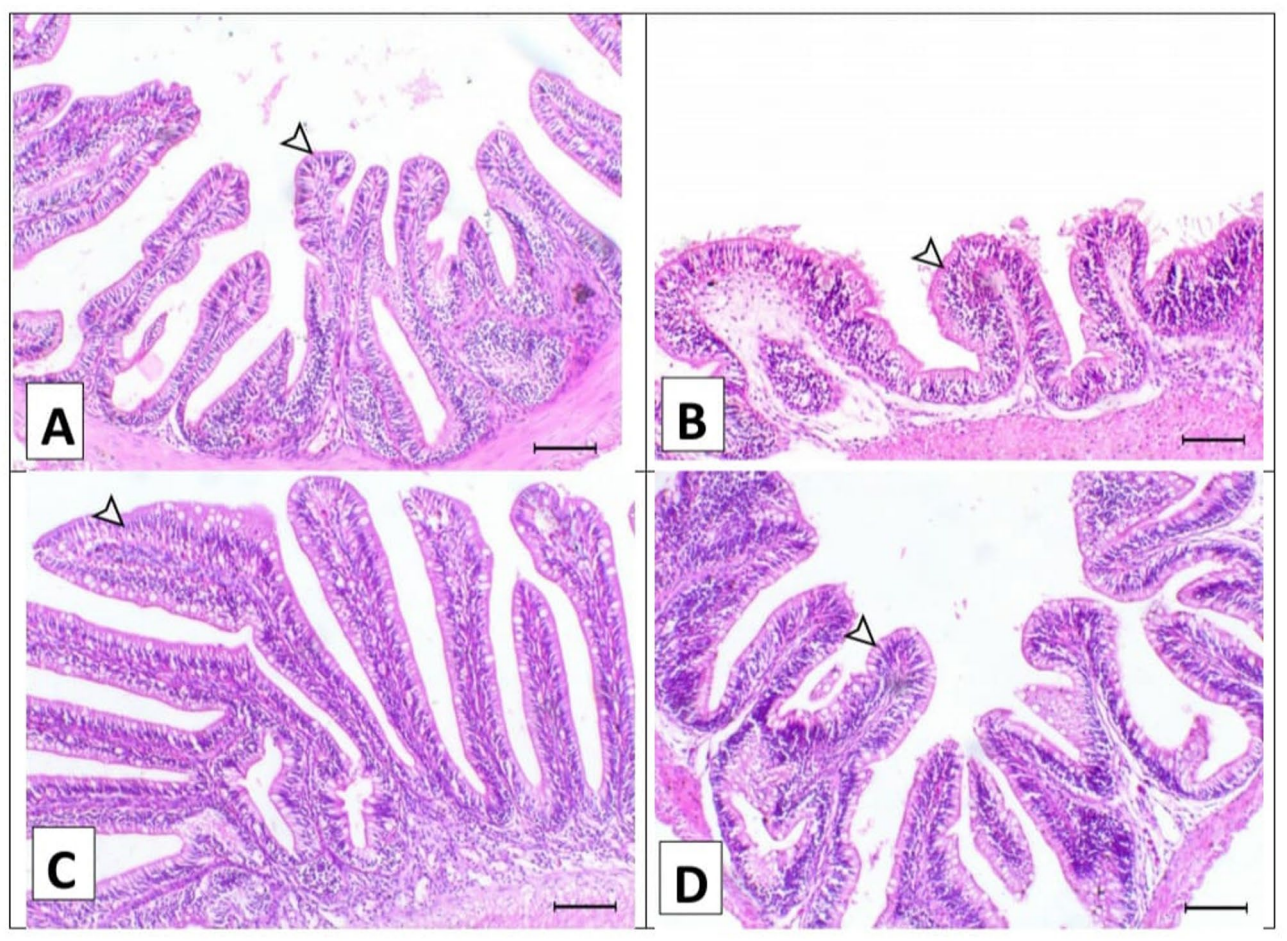
A. Intestine (middle portion) of fish supplemented with basal diet showing normal intestinal villi (arrowhead), **B**. Intestine (middle portion) of fish intoxicated with chlorpyrifos in their water showing marked blunting of the intestinal villi (arrowhead) with marked oedema within the lamina propria, **C**. Intestine (middle portion) of fish supplemented with *Artemisia Annua* in their diet showing marked increase of the intestinal villi length (arrowhead), and **D**. Intestine (middle portion) of fish intoxicated with chlorpyrifos and supplemented with *A*,* Annua* showing noticeable increase of the intestinal villi length (arrowhead). H&E, X100, bar = 100 μm.

Figure 4 showed the histopathological changes of the intestinal tissues of the various treated groups that were fed the control diet and the *A. annua* diet with/without chlorpyrifos. The middle portion of the intestine of fish supplemented with a formulated diet showed normal intestinal villi (A). Meanwhile, the middle portion of the intestine of fish intoxicated with chlorpyrifos in their water showed marked blunting of the intestinal villi with marked oedema within the lamina propria (B). The middle portion of the intestine of fish supplemented with *A. annua* in their diet showed a marked increase of the intestinal villi length (C). The middle portion of the intestine of fish intoxicated with chlorpyrifos and supplemented with *A. annua* showed a noticeable increase in the intestinal villi length (D).

The results demonstrated significant differences in the intestinal morphometrical parameters among the various groups (Table 5). The control group exhibited moderate villi length, while the chlorpyrifos-exposed group had the shortest villi length. However, the group supplemented with the *A. annua* showed an increase in the villi length^28^.

Regarding villi width, the control group and *A. annua*-supplemented group exhibited a significant increase compared to the chlorpyrifos group^29^.

In terms of goblet cell numbers, the *A. annua*-supplemented group showed a marked increase in the density of goblet cells, significantly higher than the control and other groups. Conversely, the chlorpyrifos group had the lowest goblet cell count, with the *A. annua* group showing a slight improvement compared to chlorpyrifos alone^30^.

**Table 5:** Effect of dietary *A. annua* on Intestinal Morphometry of Nile Tilapia under Chlorpyrifos Toxicity.

Groups Items	Cont	CPF	Art	CPF + Art
Villi length (µm)	431.06 ± 2.38 ^b^	251.48 ± 2.33 ^d^	620.63 ± 2.50 ^a^	382.75 ± 1.35 ^c^
Villi width (µm)	124.98 ± 2.00 ^a^	114.29 ± 1.76 ^c^	126.72 ± 2.03 ^a^	120.98 ± 1.68 ^b^
Goblet cell (No/mm2)	232.00 ± 1.00 ^b^	118.67 ± 0.88 ^d^	263.33 ± 2.73 ^a^	198.67 ± 1.86 ^c^

Values are means ± standard error. Mean values with different letters at the same row significantly differ at (*P* ≤ 0.05).

## Discussion

### Clinical signs and postmortem lesions of the chlorpyrifos toxicity

The clinical signs reported in this study, particularly the thick mucus film, abnormal movements, and neurological symptoms in CPF-exposed fish, are highly indicative of chlorpyrifos (CPF) toxicity, as documented in similar studies^31^; Amin et al.,, 2023.Moreover, Deb and Das^3^, described several behavioral and physiological anomalies, including erratic swimming and paralysis, as common symptoms following CPF exposure. These neurotoxic effects align with the observed abnormal movements and thick mucus secretion in this study, which are indicative of severe cholinergic dysfunction due to acetylcholinesterase (AChE) inhibition, a hallmark of CPF toxicity. On the other hand, the post-mortem lesions observed, including pale gills and internal organ congestion, also reflect patterns found in previous studies. Abdo^32^, described significant histopathological changes in gills and other tissues, caused by oxidative stress and immune dysfunction following CPF exposure. These findings reinforce the observation of respiratory and systemic physiological disruptions in this study, as indicated by congested organs and impaired gill function in the untreated fish. Tawfeek et al.^33^, similarly documented severe tissue damage and respiratory dysfunction in CPF-exposed fish, linking these outcomes to oxidative stress and tissue degeneration^34^.

Mortality rates were notably higher in the CPF-exposed groups in this study, particularly in the untreated fish, and these results align with earlier research. Girón-Pérez et al.^35^, reported a dose-dependent increase in mortality with CPF exposure, reaching 100% at higher doses. This supports the present findings that untreated CPF-exposed groups experienced significant mortality. Additionally, Tawfeek et al.^33^, found similarly high mortality rates, especially when CPF exposure was combined with a bacterial challenge, underscoring CPF’s ability to exacerbate physiological stress and increase mortality. The observed reduction in symptoms and mortality through the use of *A. annua* aligns with the protective effects reported in other studies using various interventions, highlighting the potential of this treatment in mitigating CPF-induced damage. For instance, *A. annua* has demonstrated significant antioxidant properties, which help mitigate oxidative stress and biochemical disruptions caused by CPF^36,37^. These findings underscore the promising role of this treatment in protecting against CPF-induced toxicity^38^.

### Growth performance

The findings of the growth performance are constant with the finding of Hossain et al.^5^,, who documented similar negative impacts, reporting reductions in specific growth rate (SGR) and survival in Nile tilapia under chlorpyrifos exposure, attributed to physiological disruptions caused by the pesticide. Conversely, the Art group (supplemented with *A. annua*) demonstrated a substantial improvement in growth metrics, surpassing all other groups. **Dai et al., (2023**) confirmed that dietary *A. annua* in fish diets supports growth and enhances antioxidant capacity, which is beneficial under stress conditions. Furthermore, **Tzortzatos et al., (2024)** reported that *A. arborescens* improved growth, immune responses, and oxidative stress markers in gilthead seabream, suggesting that *Artemisia*species are valuable feed additives for enhancing resilience against environmental contaminants like chlorpyrifos. These results, combined with studies by Francis et al.^39^,, emphasize the value of plant-based feed additives, which improve nutrient uptake and feed efficiency, reinforcing *A. annua’s* role as a growth enhancer in aquaculture. Such effects were also observed in the present study, as the Art group displayed superior feed conversion ratios (FCR)^14^.

### Biochemical parameters

The results of biochemical marker reveal that chlorpyrifos exposure significantly impairs liver function, as indicated by elevated liver enzyme activities. Chlorpyrifos, a well-known organophosphate pesticide, is hepatotoxic and can disrupt liver enzyme activity, causing oxidative damage and cellular stress. (Majumder and Kaviraj^4^ as well as Hossain et al.^5^, reported similar increases in AST and ALT activities in Nile tilapia, reflecting substantial liver damage under chlorpyrifos exposure. Girón-Pérez et al.^35^, supported these findings by demonstrating that CPF disrupts normal physiological functions in Nile tilapia, particularly affecting the immune system and phagocytic activity. On the other hand, the supplementation with *A. annua* (Art group) significantly reduced liver enzyme levels, demonstrating its hepatoprotective effect. Recent research by Soares et al., (2022) also observed that dietary *A. annua*extract improved liver function in Nile tilapia, suggesting that the bioactive compounds, including flavonoids and sesquiterpene lactones, mitigate oxidative and inflammatory stress. This aligns with the findings of He et al.^40^, who noted enhanced liver health and biochemical parameters in fish treated with *A. annua* extracts^41^.

### Gene expression

Growth hormone and insulin-like growth factor-I are considered the key genes affecting the growth that comprise the core of the hypothalamic-pituitary–somatotropic axis. Numerous factors affect these genes, including an organism’s environment, genetic makeup, and diet^42^. However, the results of the growth have been confirmed by the results of gene expression of the growth-related genes in the different treatment groups, especially the chlorpyrifos group. To our knowledge, there is no research on the extent to which the treatment used in this study affect the gene expression of growth genes. All research deals with their effect on antioxidants and immunity- related genes. In general, it has been demonstrated that polyphenols enhance the synthesis of DNA, RNA, and proteins, as well as the generation and function of *GH* and *IGF-1*, and they also promote the activity of digestive enzymes (^43,44^; Midhun et al.,, 2016)^45^.

### Histopathological finding

The gill sections from fish supplemented with either the control diet or *A. annua* demonstrated normal histological features. This indicates that *A. annua* did not induce any harmful effects when used as a dietary supplement. Dai et al., (2023) reported that fish fed with *A. annua* exhibited elongated and healthier intestinal villi, indicating improved nutrient absorption capacity and overall gut health. This aligns with reports showing that the bioactive compounds in *A. annua*, such as flavonoids and sesquiterpenes, possess strong anti-inflammatory and antioxidant properties, which help maintain the structural integrity of the intestinal lining and reduce inflammation​Septembre-Malaterre et al.,,2020^46^;.

The findings of the histopathology of the Chlo group showed worse histological changes in the gills, liver, and intestine. This supports earlier research indicating that chlorpyrifos, a widely used organophosphate pesticide, can induce significant oxidative stress and inflammation, leading to cellular damage in fish gills^32,47^**.** Moreover, chlorpyrifos was found to have worse histological changes in the liver, gills, and muscle tissues than other groups of Nile tilapia^5^. On the other side, the observed necrotic changes and leukocytic infiltration following chlorpyrifos exposure mirror findings by Pal et al.^48^,, Farhan et al., (2021), and Raibeemol & Chitra (2020), who reported similar vacuolization and necrosis in hepatic tissues of fish exposed to chlorpyrifos. Dawood et al., (2020b) reported that chlorpyrifos exposure in fish leads to severe inflammatory responses, including the sloughing off of villi tips and immune cell infiltration. Also, these findings are consistent with other studies demonstrating that chlorpyrifos induces oxidative stress and damages the intestinal lining, leading to villi shortening and compromised nutrient absorption (Mokhbatly et al.,, 2020).

However, these deleterious effects of chlorpyrifos mitigated by dietary *A. annua*. Similar studies found that *Artemisia annua* help reduce oxidative damage in fish gills exposed to pollutants, aligning with the current observation of reduced necrosis and lamellar thickening^49^. However, the moderate degenerative changes in the hepatopancreas seen with *A. annua* align with findings by Abdel-Hussein et al., (2021), who conducted a study to evaluate the therapeutic effect of *A. annua* against lead toxicity in rats. The results showed normal histology of the liver in rats given lead acetate at 15 mg/kg per day for 15 days, then treated with 100 mg/ml b.w.t of *A. annua* extract for 2 weeks. However, the current results indicate that the organs of the fish intoxicated with chlorpyrifos and supplemented with *A. annua* showed normal tissue. This difference may be due to differences in treatment dosage or the specific pollutants studied.

## Conclusion

In conclusion, this study demonstrated the detrimental effects of chlorpyrifos on the growth, biochemical marker, and pathophysiology of various organs in Nile tilapia fish. Supplementing with *A. annua* lessened these negative effects. Therefore, the protective effect of *A. annua* in the diet is demonstrated by the improvement of growth performance parameters and growth-related gene expression, biochemical markers, and histopathology of the different organs of *Oreochromis niloticus*.

## Data Availability

The datasets used and/or analyzed during the current study available from the corresponding author on reasonable request.
